# Metabotropic Glutamate Receptor Subtype 5 in Alcohol-Induced Negative Affect

**DOI:** 10.3390/brainsci9080183

**Published:** 2019-07-30

**Authors:** Chelsea R. Kasten, Eleanor B. Holmgren, Tiffany A. Wills

**Affiliations:** LSU Health Sciences Center—New Orleans, Department of Cell Biology and Anatomy, Medical Education Building, 1901 Perdido Street, Room 6103, New Orleans, LA 70112, USA

**Keywords:** alcohol, negative affect, despair, anxiety, mGlu5, sex differences, ethanol dependence

## Abstract

Allosteric modulators of metabotropic glutamate 5 receptors (mGlu_5_ receptors) have been identified as a promising treatment to independently alleviate both negative affective states and ethanol-seeking and intake. However, these conditions are often comorbid and might precipitate one another. Acute and protracted ethanol withdrawal can lead to negative affective states. In turn, these states are primary drivers of alcohol relapse, particularly among women. The current review synthesizes preclinical studies that have observed the role of mGlu_5_ receptor modulation in negative affective states following ethanol exposure. The primary behavioral assays discussed are ethanol-seeking and intake, development and extinction of ethanol-associated cues and contexts, behavioral despair, and anxiety-like activity. The work done to-date supports mGlu_5_ receptor modulation as a promising target for mediating negative affective states to reduce ethanol intake or prevent relapse. Limitations in interpreting these data include the lack of models that use alcohol-dependent animals, limited use of adolescent and female subjects, and a lack of comprehensive evaluations of negative affective-like behavior.

## 1. Introduction

Metabotropic glutamate 5 receptors (mGlu_5_ receptors) represent a viable target for the treatment of alcohol-use disorders (AUDs) and negative affective phenotypes, and their use for each of these was recently independently reviewed [1,2,3]. However, negative affective states are also known to play a role in AUDs, as they are a primary driver of drinking and relapse behaviors [4]. “Negative affect” is a defined set of negative emotional states, which underlie many highly comorbid psychiatric disorders, including depression and anxiety [5]. Preclinically, negative affective symptoms can be clustered into changes in reinforcer seeking and consumption, behavioral despair, anxiety-like activity, increased threat monitoring, hypervigilance, and home cage activity [6,7,8]. These negative affective states might heighten sensitivity to alcohol-related cues and drive relapse [9,10]. Moreover, disorders that encompass negative affect are more prevalent in women and are highly comorbid with AUDs [11,12]. The current review will synthesize research that has investigated the role of mGlu_5_ receptors in alcohol use and negative affect, as well as identify gaps in the literature, particularly in regard to sex differences. 

This review will primarily integrate the work involved in mGlu_5_ receptor modulation and its ability to regulate ethanol intake, the salience of ethanol-associated cues and contexts, and ethanol-induced behavioral despair and anxiety-like activity. mGlu_5_ and mGlu_1_ receptors comprise the group 1 metabotropic glutamate receptor class (mGlu_1/5_ receptors), which are Gα_q/11_-coupled receptors that regulate synaptic plasticity [13]. In these studies, mGlu_5_ receptors have been modulated using genetic manipulations or pharmacological tools. The role of global central nervous system knockout of mGlu_5_ receptors in drug use was first reported by Chiamulera et al. [14], who demonstrated that mGlu_5_-receptor-null mice do not acquire cocaine self-administration. Many tools beyond global knockout now exist, including cell-specific knockout [15,16], mGlu_5_ receptor deficiency [17], and mGlu_5_ receptor point mutations [18,19]. These studies used allosteric modulators, which are ligands that bind to a receptor site that is distinct from the endogenous or orthosteric ligand, as pharmacological interventions. Allosteric modulators negatively or positively regulate the activity of a receptor in the presence of its orthosteric ligand, but might also act as allosteric agonists or inverse agonists. mGlu_5_ receptor allosteric modulators discussed in the current review include the positive allosteric modulator (PAM), 3-Cyano-*N*-(1,3-diphenyl-1*H*-pyrazol-5-yl)benzamide (CDPPB), and the negative allosteric modulators (NAM), 2-Methyl-6-(phenylethynyl)pyridine (MPEP) and 3-((2-Methyl-1,3-thiazol-4-yl)ethynyl)pyridine (MTEP). CDPPB has been noted for its interaction with the MPEP binding site, as well as its ability to mediate aberrant phenotypes associated with dysregulation of the ionotropic glutamate N-methyl-D-aspartate receptor (NMDAR) [20]. It should be noted that MPEP and MTEP both act as inverse agonists to block the constitutive activities of the mGlu_5_ receptors in vivo [21,22]. They also differ in their selectivity for mGlu_5_ receptors. Although MPEP is selective for mGlu_5_ receptors at lower doses, it becomes less selective as the dose increases and acts at other receptors, including NMDARs. The more recently developed MTEP conserves selectivity for mGlu_5_ receptors without demonstrating off target activity at NMDARs [2]. All of these drugs have been implicated as potential therapeutic treatments to alleviate negative affective symptomology and drug-seeking behavior [2,20].

## 2. Ethanol Intake

The ability of mGlu_5_ receptors to modulate ethanol intake has recently been extensively reviewed [3]. Generally, treatment with mGlu_5_ receptor NAMs results in decreased ethanol consumption and responding in 24 h access 2-bottle choice, limited access, and operant drinking paradigms (see Table 1). With the exception of Adams et al. [23] systemic mGlu_5_ receptor modulation consistently reduced drinking across a range of paradigms, species, and strains. Conversely, antagonism of mGlu_1_ receptors via systemic treatment of CPCCOEt was less effective at reducing ethanol intake or resulted in off-target effects, including reduced locomotion or sucrose intake [24,25,26]. Although higher doses of mGlu_5_ receptor NAMs also reduced locomotion and sucrose intake in some studies, these doses are beyond the efficacious dose for ethanol intake [26,27,28,29]. This further indicates that mGlu_5_ receptor-targeted treatment could be well tolerated as a clinical intervention compared to mGlu_1_-receptor pharmacological interventions. Finally, systemic MPEP prevents the alcohol deprivation effect following free-choice and operant ethanol access [24,30]. However, its efficacy may be reduced over repeated deprivation cycles [24], indicating the necessity of using alcohol-dependent models. Repeated alcohol exposure and withdrawal cycles promote neuroadaptations [9,10], which might lessen the efficacy of a drug that was initially promising.

Due to the allosteric properties of CDPPB, MPEP, and MTEP, it is important to consider whether alcohol exposure affects receptor availability and how that could inform the appropriate pharmacological treatment for different populations with AUDs. Using the highly potent and selective mGlu_5_ receptor NAM ^18^[F]-FPEB in PET scans, mGlu_5_ receptor availability has been demonstrated to be relatively stable in healthy humans over a 6 month period [38]. However, higher doses of MTEP are required to reduce ethanol consumption in alcohol-dependent rats [36], and alcohol has been demonstrated to alter mGlu_5_ receptor availability in both humans and rodents. In rodents, relatively low doses of forced ethanol over a two-week period enhances striatal, hippocampal, and cortical mGlu_5_ receptor availability compared to saline controls [39]. In contrast, chronic free-choice access to ethanol decreases mGlu_5_ receptor availability in the hippocampus and amygdala, when compared to baseline levels [40]. These PET studies lend support to the hypothesis that extensive alcohol exposure shifts the availability of mGlu_5_ receptors, thereby resulting in reduced efficacy of MTEP to reduce drinking in dependent rats [36]. Similar results have been found in humans, where increased mGlu_5_ receptor availability primarily in cortical regions is associated with “feeling high” during alcohol exposure in healthy, low-drinking humans [41]. Alcohol-dependent individuals have lower mGlu_5_ receptor availability compared to controls, across many striatal and cortical regions [42]. Availability of mGlu_5_ receptors recovers in a site- and time-dependent manner, across 6 months of alcohol abstinence, except in the hippocampus, accumbens, and thalamus [43]. This reduced mGlu_5_ receptor availability in alcohol-dependent subjects may be mediated by comorbid substance use, such as smoking status [44]. Non-smoking alcohol-dependent males show increased, not decreased, mGlu_5_ receptor availability in cortical regions and the amygdala at one month of abstinence [44]. Notably, reduced mGlu_5_ receptor availability is related to increased alcohol craving, regardless of smoking status [42,43,44]. Collectively, this work points toward dynamic regulation of mGlu_5_ receptor availability across early alcohol use, chronic alcohol use, alcohol abstinence, and comorbid drug use. It is still unclear from this work if there is a causal relationship between receptor availability and excessive ethanol drinking, dependence, and craving. Genetic variants in the *mGluR-eEf2*-α-amino-3-hydroxy-5-methyl-4-isoxazolepropionic acid receptor (AMPAR) pathway, including *GRM5*, predict alcohol intake [45] and might independently regulate receptor availability. These findings complement the pharmacological studies mentioned above in pointing towards differences in mGlu_5_ receptor availability and pharmacological efficacy, depending on the type of alcohol exposure and genetic predisposition.

The studies detailed in Table 1 primarily focused on adult males; however, there is strong clinical evidence that supports the need to observe ethanol consumption in females and adolescents following mGlu_5_ receptor modulation. Adolescence is the time when alcohol use is typically initiated, and this use is known to be one of the strongest predictors for later development of AUDs [12,46]. While males are likely to use alcohol as positive reinforcement, females are more likely to use alcohol as a negative reinforcement coping mechanism [47]. Females are 2–3 times more likely to develop stress and anxiety disorders that might contribute to negative affective states, and this divergence of risk coincides with adolescent alcohol exposure [11]. It has been demonstrated that women have lower mGlu_5_ receptor availability across many brain regions [48], thereby indicating that there may be sex-differences in response to allosteric modulators. Cozzoli et al. [31] investigated the interaction of both adolescence and sex on ethanol intake. MTEP effectively reduced ethanol intake in both adolescent and adult male and female mice. However, during protracted abstinence (21 days), prior MTEP treatment showed no long-term effects on ethanol consumption in males of either age group. Females exposed to MTEP pretreatment during adolescence reduced their ethanol consumption in adulthood, whereas their adult-treated counterparts showed increased alcohol consumption. In another study, Parkitna et al. [15] demonstrated that knockdown of mGlu_5_ receptors on D1 neurons did not alter acquisition of ethanol intake under a continuous access instrumental response paradigm in females, but did inhibit an ethanol deprivation ramp-up during forced abstinence. Although males of the same strain demonstrated alcohol deprivation ramp-up of intake, it was not altered by mGlu_5_ receptor knockdown [16]. However, the male and female paradigms differed in length of alcohol history and instrumental response criteria, making it difficult to determine if the disparate findings were due to methodological- or sex-differences. Although mGlu_5_ receptor NAMs have not yet been investigated for their potential to alleviate AUD and comorbid symptomology, multiple treatments that target this system with minimal side effects have been developed for clinical use [49]. The preclinical studies indicate that alcohol duration, length of abstinence, age, and sex are all-important considerations in judging the effectiveness of mGlu_5_ receptor NAMs.

## 3. Ethanol-Associated Cues and Contexts

The role of mGlu_5_ receptors in learning and memory of discrete and contextual cues has been well documented. mGlu_5_ receptor activity contributes to neural plasticity via both long-term depression (LTD), as well as long-term potentiation (LTP) via mGlu_5_-NMDAR interactions discussed in Section 6 [13,50]. In rodent models of non-dependent ethanol intake, mGlu_5_ receptor modulation effectively reduces the salience of ethanol-associated cues and contexts when administered following the cue–ethanol association (see Table 2). The ability to modulate the salience of ethanol-associated cues and contexts, plays an important role in reducing susceptibility to relapse, making it a critical target for pharmacological intervention [51,52]. Two paradigms have been primarily used to observe the role of mGlu_5_ receptors in ethanol-associated cues and contexts—ethanol cue-induced reinstatement and conditioned place preference (CPP). 

With the exception of Adams et al. [23,57], systemic and site-specific negative allosteric modulation of mGlu_5_ receptors was found to reduce cue-induced reinstatement in the presence of discrete cues, such as a light cue [53,54,55], or diffuse contextual stimuli, such as an olfactory scent [30]. Contextual cues have been posited as more analogous to cues that induce drug craving and seeking in humans than discrete cues, due to their role in indicating general drug availability, transfer of salience, and the difficulty involved in extinguishing these cues [52]. Although Adams and colleagues [23,57] partially contributed their null findings to the low dose of MTEP used, it is important to note that their cue-induced reinstatement paradigm utilized a contextual scent to signal ethanol availability and inbred, high-alcohol-preferring iP rats. Using a discrete-cue paradigm, mGlu_5_ receptor modulation was found to readily block cue-induced reinstatement at a relatively low dose in iP rats [55]. Therefore, it might be speculated that a genetic predisposition for alcohol preference makes animals resilient to pharmacological intervention, to reduce particularly salient ethanol-associated cues. Without the investigation of higher drug doses in the iP rats, it cannot be concluded whether these disparate results were due to less sensitivity to mGlu_5_ receptor intervention under contextual cue paradigms, or whether mGlu_5_ receptors only play a role in discrete-cued reinstatement when there is a genetic predisposition to consume ethanol.

CPP, which quantifies the reinforcing value of ethanol by observing the amount of time spent in an ethanol-paired context, can be broken down into the cue/context learning phase (acquisition) and the expression of the learned association [52]. Pharmacologically or genetically reducing the activity of mGlu_5_ receptors results in impaired ethanol CPP, indicating that mGlu_5_ receptor activity contributes to the associating contexts, with ethanol. Notably, this effect appears to be restricted to the expression [25,56,58] but not acquisition of CPP [56,59]. In the acquisition studies, drug was administered prior to ethanol during the contextual-pairing sessions, whereas drug was administered without any ethanol on board during the expression test sessions. This might indicate that mGlu5 receptor modulation is not able to overcome the salience of ethanol exposure as it occurs, but rather it blocks the recall of ethanol-associated cues. 

In direct opposition to the current studies, it has been reported that mGlu_5_ receptor NAMs often inhibit the extinction of contextual and spatial memories, whereas positive modulation enhances extinction [60,61,62,63,64]. Notably, these studies consist primarily of aversive learning conditions, such as avoidance learning, startle response, and fear conditioning. Similar to the currently discussed findings on ethanol context and cues, negative mGlu_5_ receptor modulation has been shown to block context-paired locomotor conditioning to cocaine and methamphetamine CPP [65,66]. Acknowledging the divergent effects of negative mGlu_5_ receptor modulation on cues and contexts associated with positive versus negative stimuli is important. In states of dependence and withdrawal, positive ethanol-associated cues might transfer their association to negative affective states. Therefore, these once positive cues might be more similar to the aversive cues that are enhanced by negative mGlu_5_ receptor modulation. Sidhpura et al. [36] demonstrated that, although MTEP was still effective at blocking stress-induced reinstatement in dependent rats, it was more effective in non-dependent rats. mGlu_5_ receptor NAMs might also be an insufficient treatment for people experiencing an ethanol relapse. Positive mGlu5 modulation has been demonstrated to rescue impaired spatial learning following heavy ethanol exposure [67], indicating its safety and efficacy in models of dependence. Although the studies discussed within this section favor negative mGlu_5_ receptor modulation for mediating ethanol-associated cues and contexts, positive mGlu_5_ receptor modulation might be a better course of treatment under aversive states associated with alcohol withdrawal and relapse.

Females were not included in any of the discussed studies that observed cue-induced cue reinstatement. In rodents, acute pharmacological stress significantly enhances cue-induced ethanol reinstatement in females, but not males [68]. In humans, stress has not been demonstrated to enhance ethanol craving or relapse to a greater degree in females than males. However, these human studies either did not report estradiol levels or estrous status [69,70], or restricted female testing to periods when circulating estradiol levels were low [71]. In rodents, circulating estradiol levels were significantly positively correlated with the magnitude of stress-induced reinstatement [68]. Further, females demonstrate an enhanced ethanol CPP that is dependent upon circulating hormones [72], as well as alterations in drug efficacy to reduce ethanol intake based on estrous status [73]. These data indicate that circulating hormones mediate the salience of cues and stress on ethanol-associated activity and should be included in human female studies. There is support for direct interaction of mGlu_1/5_ receptors and estrogen receptors (ER) signaling (see Section 6). This convergence of signaling cascades might indicate that mGlu_5_ receptor modulation would be a particularly salient treatment in female populations.

## 4. Behavioral Despair

Behavioral despair, or anhedonic activity, is observed in animal models that represent depressive-like behavior. Although many animal models of behavioral despair exist—including reduction in intake of appetitive reinforcers, the forced swim test, the tail suspension test, social interaction, and response to novelty [8,74]—few have been observed following ethanol administration and/or mGlu_5_ receptor manipulation (see Table 3). Of these studies, the forced swim task has been the predominantly used paradigm. This task observes the time spent immobile in a container of water. Time spent immobile is decreased by treatment with antidepressants, indicating translational relevance [74]. Limited access ethanol in adult male mice reliably induces behavioral despair in the forced swim test 24 h into withdrawal [75,76,77]. This effect is rescued by systemic MTEP treatment, but not site-specific treatment targeting the NAc shell [75,76]. Conversely, systemic treatment with the mGlu_5_ receptor PAM, CDPPB, exacerbates the effect of ethanol withdrawal on behavioral despair [75]. Adolescent ethanol exposure, however, does not result in a behavioral despair phenotype in male mice 24 h into withdrawal. In contrast, CDPPB administration is able to induce a behavioral despair phenotype in water drinking controls [75]. Interestingly, protracted withdrawal from adolescent alcohol does result in a behavioral despair phenotype [76], but it is not known if it can be rescued via systemic mGlu_5_ receptor modulation. 

Although mGlu_5_ receptors appear to be a promising target for rescuing behavioral despair induced by ethanol exposure, these studies suffer from lack of diversity in tests, ethanol exposure paradigms, sex, and age. All studies discussed in this section used a 14-day drinking-in-the-dark exposure in male B6 mice. No studies have observed the effects of ethanol dependence, prolonged ethanol exposure, or protracted ethanol withdrawal in adulthood on behavioral despair. These studies are necessary to accurately capture the ability of mGlu_5_ receptor modulation to mediate the negative-affective withdrawal phenotype that promotes relapse susceptibility. Male and female rodents also express behavioral despair in a sex-dependent manner. For example, females are susceptible to the forced swim test, but relatively resilient to psychosocial models of despair [74]. The forced swim task suffers from many criticisms, which include the lack of translational value for treatment development, dependence on physical activity, and differing survival strategies to conserve energy [74,78]. However, the forced swim test is high-throughput, and engages overlapping neural circuitry with humans suffering from depression [74], making it a valuable tool when paired with other behavioral despair tests. Complementary tasks may include seeking and consumption of non-drug reinforcers, tail suspension task, social interaction, response to novelty, and observation of normative home-cage activities such as grooming [6,8]. As these tests result in sexually distinct phenotypes that are not consistent between tasks [8], it is important to include multiple behavioral paradigms to observe how ethanol alters the complete behavioral despair phenotype. Finally, as mGlu_5_ receptor modulation shows promise in these studies, it should be examined at both younger and older ages, following alcohol exposure, dependence, and protracted withdrawals. Depression at both young and old age is associated with poor outcomes and limited response to traditional antidepressants [74], making the mGlu_5_ receptors a promising target.

mGlu_5_ receptors have been extensively implicated in major depression disorder (MDD) at the clinical and preclinical levels, as recently reviewed by Esterlis et al. [79]. Similar to the studies discussed that have observed mGlu_5_ receptor availability in alcohol use disorders (see Section 2), studies observing those with MDD without alcohol and substance use disorders have reported mixed findings. One study has reported increased post-mortem *Grm5* expression in the locus coeruleus of MDD individuals, noting the important role of locus coeruleus excitability in MDD [80]. Studies reporting reduced mGlu_5_ receptor availability in those with MDD have been conducted in non-smoking populations [81,82], whereas those that reported no differences overwhelmingly included smoking individuals [83,84,85,86]. Smoking status also mediates the relationship between mGlu_5_ receptor availability and alcohol use, but it appears that smoking is responsible for the reduced mGlu_5_ receptor availability in heavy drinkers [44]. Although the relationships between mGlu_5_ receptor availability and smoking status in heavy alcohol use and MDD are divergent, it is still notable that smoking status might alter response to mGlu_5_ receptor modulators as behavioral treatments in each of these populations. Ketamine, which was recently approved by the FDA for treatment-resistant MDD, rapidly reduces availability of mGlu_5_ receptors in non-smokers with MDD and in healthy controls. The magnitude of reduction of receptor availability in the hippocampus was positively correlated with a reduction in symptoms of depression [81]. This relationship between mGlu_5_ receptor availability and depression symptomology has also been reported in non-smoking individuals not treated with ketamine [82]. Although the effects of ketamine have been linked to mGlu_5_ receptors, directly targeting mGlu_5_ receptors with NAMs has not been demonstrated to treat symptoms of MDD in clinical trials [87,88]. Notably, the primary outcome of these studies was the Montgomery–Åsberg Depression Rating Scale (MADRS), which is clinician-rated during an interview session. However, when patients are asked to self-report, negative mGlu_5_ receptor modulation significantly improves depressive symptomology and quality of life when paired with a traditional antidepressant [88]. These results indicate that treatment with mGlu_5_ receptor modulators might alleviate internal feelings of depressive symptomology that promote excessive alcohol intake. 

## 5. Anxiety-Like Activity

Anxiety-like activity is a major component of negative affective behavior, as well as a primary driver of stress and stress-induced relapse [5,10]. Several studies have observed the ability of mGlu_5_ receptor modulation to alter alcohol-induced unconditioned anxiety-like behavior across a range of paradigms. These paradigms include approach–avoidance conflict tasks (elevated plus maze, light/dark box, and the open field task) [89], as well as the marble burying task. Although marble burying is poorly correlated with traditional measures of anxiety, it is regarded as a perseverative, investigative activity that can be pharmacologically manipulated [90,91]. With few exceptions [58,75], all papers detailed in Table 4 observed increases in anxiety-like activity following ethanol exposure, which were overwhelmingly rescued by mGlu_5_ receptor NAM administration.

The efficacy of mGlu_5_ receptor NAMs to reduce heightened anxiety-like activity is consistent across ethanol i.p. administration [92], ethanol liquid diet [93], and free-choice limited ethanol access [75,76]. Within adult animals, the findings were also consistent across behavioral assays. This is notable due to the poor predictive validity of each of these tests on their own [89]. Further, in the absence of alcohol, mGlu_5_ receptor modulation is a promising target for treatment of anxiety disorders. A majority of reports using mGlu_5_ receptor modulation to alter anxiety-like activity report anxiolytic responses, whereas serotonergic, endocannabinoid, neuropeptide, and other glutamatergic targets often report inactivity of the tested compounds, or even anxiogenic activity [94,95]. In these studies, mGlu_5_ receptor modulation had minimal effects on anxiety-like activity in control mice, contrary to its predominately anxiolytic profile in many assays [95]. One reason for this might be that these tests employed parameters that evoked low baseline anxiety levels (such as low light intensity) to be able to detect heightened anxiety-like activity present in alcohol exposed mice. In typical anxiety-like assays, ceiling levels of anxiety are often provoked by bright lights, aversive or threatening stimuli, or conflict [95]. As such, the current studies indicate that targeting mGlu_5_ receptors might normalize maladaptive behavior that is present following alcohol use, without disrupting normal system function.

Although the studies examining anxiety-like activity use a wide range of ethanol exposures and behavioral outcomes, they still suffer from limitations of age and sex, with adult males being the primary demographic studied. With the exception of Lee et al. [75], mGlu_5_ receptor modulation was able to rescue anxiety-like phenotypes observed within 48 h of the last ethanol vapor. Lee et al. [75] were unable to demonstrate an enhanced anxiety-like profile in adolescent males following ethanol exposure. However, it has been well-documented that alcohol exposure during adolescence kindles anxiety-like behavior during protracted withdrawal, as mice age into adulthood [75,96,97,98,99,100,101]. In the context of preventing negative-affect-induced relapse, it is necessary to investigate whether mGlu_5_ receptor modulation might also rescue enhanced anxiety-like activity that occurs during extensive ethanol abstinence. Similarly, females may be especially sensitive to anxiety during periods of abstinence [11,47], with protracted withdrawal from adolescent alcohol resulting in enhanced anxiety-like and despair behavior [99,102]. These results highlight the need to observe the ability of mGlu_5_ receptor modulation to alter anxiety-like activity in males and females during protracted withdrawal from alcohol.

## 6. Synaptic Plasticity

While the studies discussed up to this point have focused on the behavioral outcomes of mGlu_1/5_ receptor activation, much is also known about the impacts of the cellular mechanisms of these receptors by drugs of abuse and negative affect. mGlu_1/5_ receptors are located postsynaptically or perisynaptically and are anchored to the postsynaptic density by interactions with Homer and SHANK. While mGlu_1/5_ receptors are key regulators of excitatory synaptic plasticity through both LTD and LTP, the following discussion focuses on mGlu1/5 regulation of LTD. Generally in regions like the bed nucleus of the stria terminalis (BNST) and striatum, mGlu_1/5_ receptor activation leads to phospholipase C (PLC) enhancement of PIP_2_, which in turn activates two divergent downstream pathways. One involves the activation of IP_3_ pathway and release of endoplasmic reticulum Ca^2+^ subsequent protein kinase C (PKC), mitogen-activated protein kinase kinase (MEK), and Erk1/2 activation, which can ultimately activate Arc. Secondly, activation of the IP_3_ pathway produces diacyl-glycerol (DAG), which is then acted upon by PLC and DAG lipase (DAGL) to produce the endocannabinoid, 2-AG. In LTD, the initial (early) phase of this LTD is initiated by generation of 2-AG, which is released from the postsynaptic neuron and activates presynaptic type 1 cannabinoid receptors (CB1 receptor). The activation of presynaptic CB1 receptors produces a reduction in glutamate release or an enhancement of GABA release. The maintenance (late) phase of this LTD is initiated through the actions of the IP3 pathway (discussed above) that ultimately produces the internalization of AMPARs [13]. The reliance on a raise in postsynaptic Ca^2+^ and subsequent activation of PKC or PLC, as well as the involvement of an endocannabinoid signaling, differ by brain region (reviewed in [13]). Additionally, the mode of LTD induction [drug induced via (S)-3, 5-dihydroxyphenylglycine (DHPG), paired-pulse-induces, or frequency-induced] is also thought to influence the reliance on certain mechanisms (reviwed in [13]). Further the anatomical contributions of mGlu_1_ receptors versus mGlu_5_ receptors differ by brain region. 

The extended amygdala is a collection of brain structures including the nucleus accumbens shell (NAc shell), the bed nucleus of the stria terminalis (BNST), and the central nucleus of the amygdala (CeA). These brain structures are known to play critical roles in in the modulation of negative affect and stress, particularly in the context of withdrawal [4]. In the BNST of male mice, mGlu_1/5_ receptor-mediated LTD is disrupted by chronic cocaine during both acute withdrawal and prolonged abstinence [103,104]. This cocaine-induced mGlu_1/5_ receptor-mediated LTD disruption is manifested by internalization of GluA2-containing AMPARs (calcium-impermeable), followed by replacement with calcium-permeable-AMPARs in the ventral tegmental area (VTA) and NAc [105,106,107,108]. Outside the extended amygdala, a similar disruption of mGlu_1/5_ receptor-mediated LTD is also found in the hippocampus during acute withdrawal from chronic ethanol vapor in male mice. In the CeA, there is a role for mGlu_1/5_-Homer signaling on ethanol binge-drinking [34]. A large body of literature from the Szumlinski lab finds that mGlu_1/5_ receptor signaling effects on ethanol are mediated through the interaction with Homer 2 [18,34,109,110,111,112,113]. Recently, this same group expanded on this work to find that Erk phosphorylation enhances mGlu_5_-Homer interaction in the BNST and this action attenuates ethanol drinking [19]. 

This mGlu_5_ receptor signaling mechanism was also found to be critical for estradiol-driven potentiation of psychostimulant-induced behaviors in female rodents [114,115]. Estradiol activates ERα through activation of mGlu_1/5_ receptors, thereby activating CREB and phosphorylated PLC through MAPK, independent of glutamate activation. Estradiol-induced CREB phosphorylation is differentially mediated depending on the brain region. mGlu_5_ receptor-dependent regions include the dorsal striatum and NAc core, whereas the NAc shell is mGlu_1a_ receptor-dependent. Estradiol’s interactions with mGlu_1/5_ receptors also site-specifically alters brain morphology, decreasing dendritic spines in the NAc core while increasing dendritic spines in the NAc shell. Rodent models of chronic cocaine use have demonstrated that females have fast acquisition, enhanced escalation, and greater reinstatement. ER/mGlu receptor signaling is thought to be responsible for a majority of the sex differences in cocaine behaviors and the neural transmission cocaine phenotypes elicited in females [116]. Given the efficacy of mGlu_5_ receptor modulation in males and the role of female sex hormones contributing to these behaviors in the cocaine literature, it might be expected that females would demonstrate enhanced ethanol intake, stronger associations with ethanol-associated cues and contexts, and enhanced behavioral despair and anxiety that would be particularly responsive to mGlu_5_ receptor modulation.

## 7. Conclusions

The data currently reviewed indicate that mGlu_5_ receptor modulation is a promising target for negative affect-like behavior associated with alcohol use disorders. mGlu_5_ receptor modulation is able to reduce ethanol intake, salience of ethanol-associated cues and contexts, behavioral despair, and anxiety-like activity. In humans, alcohol consumption manifests in many ways. This includes no alcohol use, light and recreational use, and dangerous levels of binging and intoxication. Currently, the literature suggests that mGlu_5_ receptors contribute to sex-specific neuroadaptations following alcohol use. These adaptations appear to be dependent upon age of onset of use, frequency of use, length of use, and length of abstinence from ethanol. Although the current studies touch on these points, the field is ripe for investigation of sex-differences, adolescent alcohol exposure, the role of alcohol dependence, and the effect of varying periods of withdrawal on the interaction of negative affect with alcohol intake and seeking. In particular, females and those exposed to alcohol during adolescence might be particularly susceptible to developing these negative affective states following protracted withdrawal from ethanol due to the role of developmental sex hormones in neuroadaptations underlying mGlu_5_ receptor signaling. The current studies also primarily focused on negative affective states following ethanol exposure. However, negative affective states often precipitate relapse. Therefore, future studies are needed to observe whether mGlu_5_ receptor modulation during periods of negative affect, such as chronic or unpredictable stressors, might work to alleviate ethanol intake. Finally, studies should consider using more than one task to observe behavioral despair and anxiety-like activity, as these phenotypes might manifest differently based on sex, age of exposure, and length of exposure or withdrawal. Although further work is required to broadly ensure the safety and efficacy of mGlu_5_ receptor modulation, the current work supports this system as a promising target for treating both ethanol-induced negative affect, as well as preventing negative affect-induced relapse. 

## Figures and Tables

**Table 1:** Details from studies on the effects of mGlu5 receptor modulation on ethanol intake in continuous access, limited access, and operant ethanol drinking paradigms.

**Continuous Access**							
**Manipulation**	**Average Reported Ethanol Intake**	**Treatment Details**	**Species/Strain/Sex**	**Housing**	**Effect**	**Dose**	**Reference**
*GRM5* mutation	TS/TS: greater than 6.0 g/kg		Male & female *GRM5^TS/TS, TS/AA, AA/AA^* mice	Grouped	Increased	AA/AA	[19]
MTEP	Up to 20 g/kg	Repeated systemic prior to access	Female B6 mice	Individual	Increased & Decreased	20 mg/kg	[31]
mGlu_5_ receptor deficiency	Wild type: greater than 9.0 g/kg		Male Grm5^tm1Rod^ mice		Decreased	n/a	[17]
mGlu_5_ receptor knockout	Wild type: up to 3.0 g/kg		Female mGlu5^−/−^ mice		Decreased, no change	n/a	[32]
MTEP	Up to 5 g/kg	Repeated systemic	Male FH rats		Decreased	2 mg/kg	[27]
MTEP	Up to 15 g/kg	Repeated systemic prior to access	Male B6 mice	Individual	Decreased	20 mg/kg	[31]
MPEP	0.53 ± 0.05 g/kg	Repeated systemic	Male Wistar rats	Individual	Decreased	3, 10 mg/kg	[30]
MPEP	Greater than 5.0 g/kg	Repeated systemic	Meyers rats	Individual	Decreased	1, 3 mg/kg	[33]
MPEP	17.9 ± 8.2 g/kg	Repeated systemic	Male B6 mice	Individual	Decreased	10 mg/kg	[25]
mGlu_5_ receptor knockdown on D1 neurons	Up to 6 g/kg		Male mGlu5^KD−D1^ mice	Individual	No change	n/a	[16]
mGlu_5_ receptor knockout	Wild type: up to 2.0 g/kg		Male mGlu5^−/−^ mice		No change	n/a	[32]
Impaired mGlu5/Homer interaction	Wild type: 10.84 ± 2.26 g/kg		Male mGlu5-F1128R mice	Grouped	No change	n/a	[18]
**Limited Access**							
**Manipulation**	**Average Reported Ethanol Intake**	**Treatment Details**	**Species/Strain/Sex**	**Housing**	**Effect**	**Dose**	**Reference**
mGlu_5_ receptor knockout	Wild type: greater than 2.0 g/kg		Female mGlu5^−/−^ mice		Decreased	n/a	[32]
MTEP	Up to 3 g/kg	Repeated systemic	Female B6 mice	Individual	Decreased	20 mg/kg	[31]
MTEP	Up to 3.5 g/kg	Repeated systemic	Male B6 mice	Individual	Decreased	10, 20 mg/kg	[31]
MTEP	Up to 4.5 g/kg	Acute intra-CeA	Male B6 mice	Individual	Decreased	3 µg/side	[34]
MPEP	Up to 1.5 g/kg	Acute intra-NAc	Male B6 mice	Individual	Decreased	0.1, 0.3, 1 µg/side	[18]
mGlu_5_ receptor knockout	Wild type: up to 1.5 g/kg		Female mGlu5^−/−^ mice		No change	n/a	[32]
MTEP	Up to 2.0 g/kg	Acute intra-adBNST	Male & female *GRM5^TS/TS, TS/AA, AA/AA^* mice	Individual	No change	30 µg/side	[19]
MPEP	Greater than 0.75 g/kg	Acute intra-NAc	Male mGlu5-F1128R mice	Individual	No change	1 µg/side	[18]
**Operant Responding**							
**Manipulation**	**Average Reported Ethanol Intake**	**Treatment Details**	**Species/Strain/Sex**	**Housing**	**Effect**	**Dose**	**Reference**
*GRM5* mutation	TS/TS: up to 1.5 g/kg		Male & female *GRM5^TS/TS, TS/AA, AA/AA^* mice	Grouped	Increased	AA/AA	[19]
mGlu_5_ receptor knockdown on D1 neurons	Wild type: up to 3000 licks		Female mGlu5^KD−D1^ mice	Grouped	Decreased	n/a	[15]
MTEP	Up to 80 responses	Acute systemic	Male FH rats		Decreased	2 mg/kg	[27]
MTEP	Greater than 100 responses	Acute systemic	Male iP rats		Decreased	1, 2 mg/kg	[27]
MTEP	Greater than 100 responses	Acute systemic	Male B6 mice	Grouped	Decreased	20, 40 mg/kg	[28]
MTEP	Up to 20 responses	Acute intra-NAc shell	Male Wistar rats		Decreased	1.5 µg/side	[35]
MTEP	Non-dependent: up to 30 responses Dependent: up to 40 responses	Acute systemic	Male Wistar rats	Grouped	Decreased	1, 3 mg/kg	[36]
MPEP	Up to 80 responses	Acute systemic	Male iP rats	Individual	Decreased	3, 10 mg/kg	[24]
MPEP	Greater than 8.0 g/kg	Acute systemic	Male B6 mice	Individual	Decreased	3, 10 mg/kg	[25]
MPEP	Up to 5 g/kg	Acute systemic	Male B6 mice		Decreased	3, 10 mg/kg	[26]
MPEP	Greater than 0.6 g/kg	Acute systemic	Male iP rats	Pair	Decreased	3, 10 mg/kg	[29]
MPEP	0.96 ± 0.22 g/kg	Acute intra-NAc medial core	Male iP rats	Individual	Decreased	10 µg/side	[37]
MTEP	Up to 15 responses	Acute intra-NAc core	Male Wistar rats		No change	1.5 µg/side	[35]
MTEP	0.60 ± 0.1 g/kg	Acute systemic	iP rats	Pair	No change	2.5 mg/kg	[23]
MPEP	1.15 ± 0.18 g/kg	Acute intra-dorsomedial caudate	Male iP rats	Individual	No change	1, 3, 10 µg/side	[37]
MPEP	1.02 ± 0.08 g/kg	Acute intra-medial PFC	Male iP rats	Individual	No change	1, 3, 10, 30 µg/side	[37]

wild-type (GRM5^TS/TS^), heterozygous mutant (GRM5^TS/AA^), homozygous mutant (GRM5^AA/AA^), C57Bl/6J (B6), Fawn Hooded (FH), central amygdala (CeA), nucleus accumbens (NAc), anterior dorsal bed nucleus of the stria terminalis (adBNST), inbred alcohol-preferring (iP), prefrontal cortex (PFC), 2-Methyl-6-(phenylethynyl)pyridine (MPEP) and 3-((2-Methyl-1,3-thiazol-4-yl)ethynyl)pyridine (MTEP).

**Table 2:** Details from studies observing the effect of mGlu5 receptor modulation on cue-induced reinstatement to seek ethanol and ethanol-conditioned place preference.

**Ethanol Cue-Induced Reinstatement**							
**Manipulation**	**Average Reported Ethanol Intake**	**Treatment Details**	**Species/Strain/Sex**	**Housing**	**Effect**	**Dose**	**Reference**
CDPPB	Up to 80 responses	Repeated systemic, during extinction	Male Wistar rats	Individual	Decreased	20 mg/kg	[53]
MTEP	Greater than 60 responses	Acute intra-BLA, prior to reinstatement test	Male Wistar rats	Individual	Decreased	3.0 µg/µl	[54]
MTEP	Greater than 40 responses	Acute intra-NAc core, prior to reinstatement test	Male Wistar rats	Individual	Decreased	3.0 µg/µl	[54]
MPEP	Up to 60 responses	Acute systemic, prior to reinstatement test	Male iP rats	Pair	Decreased	1, 10 mg/kg	[55]
MPEP	0.53 ± 0.05 g/kg	Acute systemic, prior to reinstatement test	Male Long Evans rats	Pair	Decreased	3, 10 mg/kg	[30]
MPEP	2.0 g/kg	Acute systemic, prior to reinstatement test	Male B6 mice	Grouped	Decreased	20 mg/kg	[56]
MTEP	0.54 ± 0.04 g/kg	Acute systemic, prior to reinstatement test	iP rats	Pair	No change	2.5 mg/kg	[57]
MTEP	0.60 ± 0.1 g/kg	Acute systemic, prior to reinstatement test	iP rats	Pair	No change	2.5 mg/kg	[23]
**Ethanol Conditioned Place Preference**							
**Manipulation**	**Ethanol Dose**	**Treatment Details**	**Species/Strain/Sex**	**Housing**	**Effect**	**Dose**	**Reference**
*GRM5* mutation	1.0–3.0 g/kg		Male & female *GRM5^TS/TS, TS/AA, AA/AA^* mice	Grouped	Increased Decreased	TS/TS AA/AA	[19]
mGlu_5_ receptor deficiency	1.0 g/kg		Male Grm5^tm1Rod^ mice		Decreased	n/a	[17]
MTEP	0.5 g/kg	Acute systemic, prior to test	Male Wistar rats	Grouped	Decreased	2.5, 5 mg/kg	[58]
MPEP	2.0 g/kg	Acute systemic, prior to test	Male B6 mice	Grouped	Decreased	20 mg/kg	[56]
MPEP	2.0 g/kg	Acute systemic, prior to test	Male B6 mice	Individual	Decreased	10 mg/kg	[25]
mGlu_5_ receptor knockdown on D1 neurons	1.5 g/kg		Male & female mGlu5^KD−D1^ mice	Grouped	No change	n/a	[15]
MPEP	2.0 g/kg	Repeated systemic, during acquisition	Male B6 mice	Grouped	No change	5, 10, 20 mg/kg	[56]
MPEP	2.0 g/kg	Repeated systemic, during acquisition	Male D2 mice	Grouped	No change	1, 5, 20 mg/kg	[59]

Basolateral amygdala (BLA), nucleus accumbens (NAc), inbred preferring rat (iP), C57Bl/6J (B6), DBA/2J (D2), 3-Cyano-N-(1,3-diphenyl-1H-pyrazol-5-yl)benzamide (CDPPB).

**Table 3:** Details of the effects of mGlu5 receptor modulation on behavioral despair in the forced swim task.

Manipulation	Average Reported Ethanol Intake	Treatment Details	Species/Strain/Sex	Housing	Alcohol × Drug Effect	Dose	Reference
MTEP	4.00 ± 0.05 g/kg	Acute systemic	Adult male B6 mice	Grouped	Rescued	30 mg/kg	[77]
MTEP	Greater than 4.0 g/kg	Acute systemic	Adult male B6 mice	Grouped	Rescued	30 mg/kg	[75]
CDPPB	Greater than 4.0 g/kg	Acute systemic	Adult male B6 mice	Grouped	Exacerbated	30 mg/kg	[75]
MTEP	Greater than 5.0 g/kg	Acute systemic	Adolescent male B6 mice	Grouped	No change	30 mg/kg	[75]
MTEP	Up to 7.0 g/kg	Acute intra-NAc shell	Adolescent male B6 mice	Grouped	No change	1, 10 µg/side	[76]
MTEP	Up to 5.0 g/kg	Acute intra-NAc shell	Adult male B6 mice	Grouped	No change	1, 10 µg/side	[76]
CDPPB	Greater than 5.0 g/kg	Acute systemic	Adolescent male B6 mice	Grouped	No change	30 mg/kg	[75]

C57Bl/6J (B6), nucleus accumbens (NAc).

**Table 4:** Details from studies assessing anxiety-like activity following mGlu5 modulation in the elevated plus maze (EPM), light/dark box (LD), open field (OF), and marble burying (MB) tasks.

Manipulation	Task	Average Reported Ethanol Intake	Treatment Details	Species/Strain/Sex	Housing	Alcohol × Drug Effect	Dose	Reference
MTEP	EPM	Up to 2 g/kg	Acute systemic	Adult male Wistar rats	Grouped	Rescued	2.5, 5 mg/kg	[92]
MPEP	EPM	Greater than 10.0 g/kg	Acute systemic	Male Wistar rats	Individual	Rescued	2.5, 5, 10, 20, 30 mg/kg	[93]
MTEP	LD	Greater than 4.0 g/kg	Acute systemic	Adult male B6 mice	Grouped	Rescued	30 mg/kg	[75]
MTEP	LD	Up to 5.0 g/kg	Acute intra-NAc shell	Adult male B6 mice	Grouped	Rescued	1 µg/side	[76]
CDPPB	LD	Greater than 4.0 g/kg	Acute systemic	Adult male B6 mice	Grouped	Exacerbated	30 mg/kg	[75]
MTEP	LD	Greater than 5.0 g/kg	Acute systemic	Adolescent male B6 mice	Grouped	No change	30 mg/kg	[75]
CDPPB	LD	Greater than 5.0 g/kg	Acute systemic	Adolescent male B6 mice	Grouped	No change	30 mg/kg	[75]
MTEP	LD	Up to 7.0 g/kg	Acute intra-NAc shell	Adolescent male B6 mice	Grouped	No change	1, 10 µg/side	[76]
MPEP	OF	Greater than 10.0 g/kg	Acute systemic	Male Wistar rats	Individual	Rescued	2.5, 5, 10 mg/kg	[93]
MTEP	MB	Greater than 4.0 g/kg	Acute systemic	Adult male B6 mice	Grouped	Rescued	30 mg/kg	[75]
MTEP	MB	Up to 5.0 g/kg	Acute intra-NAc shell	Adult male B6 mice	Grouped	Rescued	1 µg/side	[76]
MTEP	MB	Up to 7.0 g/kg	Acute intra-NAc shell	Adolescent male B6 mice	Grouped	Rescued	10 µg/side	[76]
MTEP	MB	Greater than 5.0 g/kg	Acute systemic	Adolescent male B6 mice	Grouped	Decreased	30 mg/kg	[75]
CDPPB	MB	Greater than 5.0 g/kg	Acute systemic	Adolescent male B6 mice	Grouped	Increased	30 mg/kg	[75]
CDPPB	MB	Greater than 4.0 g/kg	Acute systemic	Adult male B6 mice	Grouped	No change	30 mg/kg	[75]

Elevated plus maze (EPM), light/dark box (LD), C57Bl/6J (B6), nucleus accumbens (NAc), open field (OF), marble burying (MB).
